# Exploiting Heparan Sulfate Proteoglycans in Human Neurogenesis—Controlling Lineage Specification and Fate

**DOI:** 10.3389/fnint.2017.00028

**Published:** 2017-10-17

**Authors:** Chieh Yu, Lyn R. Griffiths, Larisa M. Haupt

**Affiliations:** Genomics Research Centre, Institute of Health and Biomedical Innovation, School of Biomedical Sciences, Queensland University of Technology, Brisbane, QLD, Australia

**Keywords:** heparan sulfate proteoglycan, stem cell, neurogenesis, syndecan, glypican, perlecan, growth factors, biomimetics

## Abstract

Unspecialized, self-renewing stem cells have extraordinary application to regenerative medicine due to their multilineage differentiation potential. Stem cell therapies through replenishing damaged or lost cells in the injured area is an attractive treatment of brain trauma and neurodegenerative neurological disorders. Several stem cell types have neurogenic potential including neural stem cells (NSCs), embryonic stem cells (ESCs), induced pluripotent stem cells (iPSCs), and mesenchymal stem cells (MSCs). Currently, effective use of these cells is limited by our lack of understanding and ability to direct lineage commitment and differentiation of neural lineages. Heparan sulfate proteoglycans (HSPGs) are ubiquitous proteins within the stem cell microenvironment or niche and are found localized on the cell surface and in the extracellular matrix (ECM), where they interact with numerous signaling molecules. The glycosaminoglycan (GAG) chains carried by HSPGs are heterogeneous carbohydrates comprised of repeating disaccharides with specific sulfation patterns that govern ligand interactions to numerous factors including the fibroblast growth factors (FGFs) and wingless-type MMTV integration site family (Wnts). As such, HSPGs are plausible targets for guiding and controlling neural stem cell lineage fate. In this review, we provide an overview of HSPG family members syndecans and glypicans, and perlecan and their role in neurogenesis. We summarize the structural changes and subsequent functional implications of heparan sulfate as cells undergo neural lineage differentiation as well as outline the role of HSPG core protein expression throughout mammalian neural development and their function as cell receptors and co-receptors. Finally, we highlight suitable biomimetic approaches for exploiting the role of HSPGs in mammalian neurogenesis to control and tailor cell differentiation into specific lineages. An improved ability to control stem cell specific neural lineage fate and produce abundant cells of lineage specificity will further advance stem cell therapy for the development of improved repair of neurological disorders. We propose a deeper understanding of HSPG-mediated neurogenesis can potentially provide novel therapeutic targets of neurogenesis.

**Graphical Abstract:**
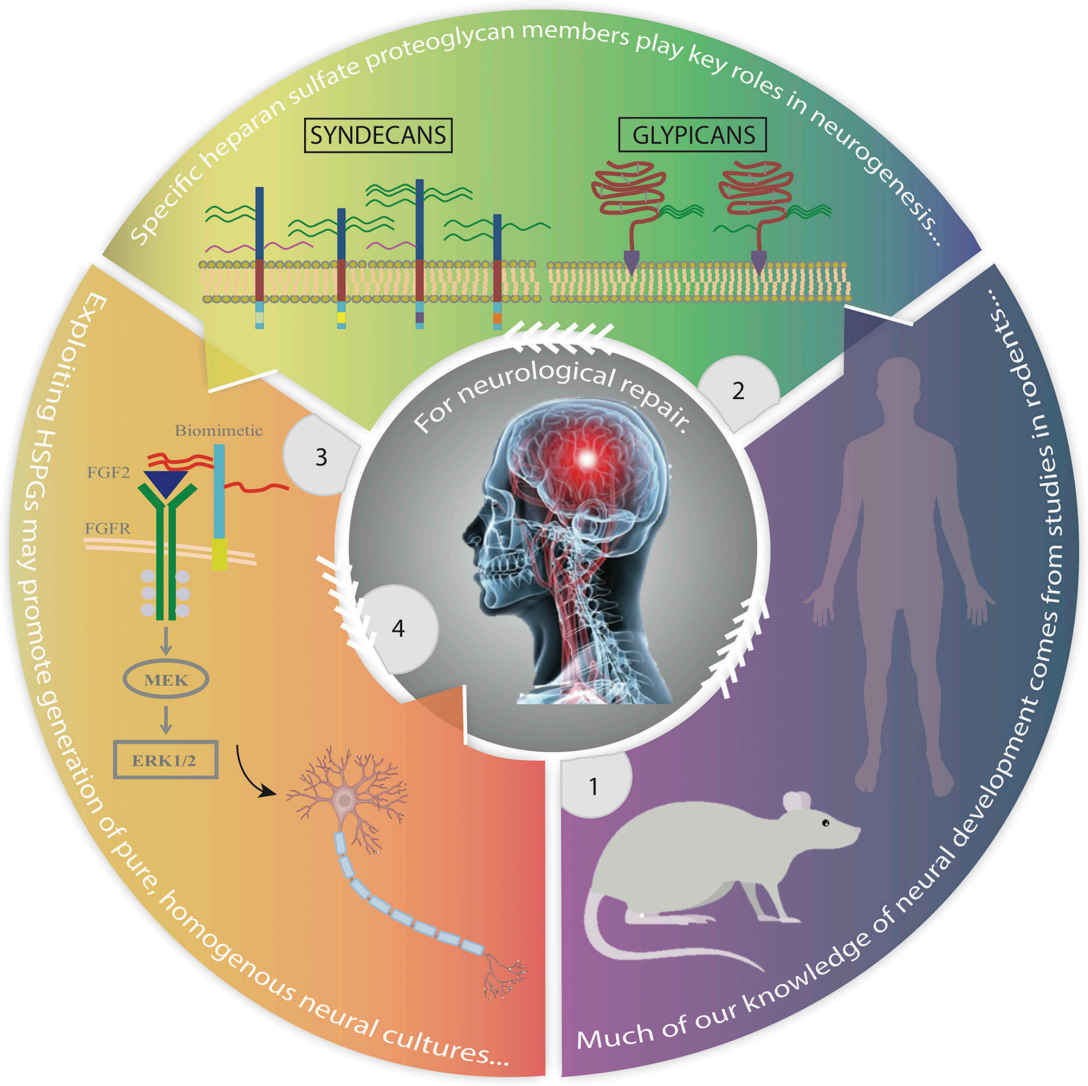
Advances in the study of mammalian neural development identifying heparan sulfate proteoglycans as key regulators that may be exploited to improve stem cell replacement therapy. **Panel 1**. Most of our current knowledge of human neural development is derived from studies in rodents. **Panel 2**. HSPGs, particularly membrane-bound syndecans and glypicans, are major regulators of neurogenesis. **Panel 3**. Exploiting the role of HSPGs in mediating stem cell fate through use of biomimetics, growth factor-receptor relationships and signaling pathways, we can develop tailored high yields of lineage specific neural cells. **Panel 4**. This strategy provides promise for the treatment and repair of neurological disorders. Rat image obtained from: http://www.transposagenbio.com/hubfs/products/tpb-ProductImage-Rat-02.png?t=1487023367000; human image obtained from: https://commons.wikimedia.org/wiki/File:Human_body_silhouette.svg.

## Key concepts

1. Stem cells are unspecialized cells capable of self-renewal and differentiation into specialized cell types under appropriate conditions.
2. Neurogenesis is defined as the generation of functional neurons from neural precursors and is the first step in neural development, followed by axon guidance and synaptogenesis.
3. Neural stem cells, embryonic stem cells, induced pluripotent stem cells, and mesenchymal stem cells are all capable of differentiating into neural cell types.
4. Neurological disorders, including traumatic brain injuries and neurodegenerative diseases are commonly characterized by the loss of neural cells—current treatments provide some symptomatic improvement thus stem cell therapy in the form of cell/tissue replacement is a promising alternative.
5. Heparan sulfate proteoglycans (HSPGs) are ubiquitous molecules on the cell surface and in the extracellular matrix which act as intermediaries between the extracellular matrix and intracellular signaling pathways, playing critical roles in numerous cellular activities.
6. Syndecans, glypicans, and perlecan HSPGs carry heparan sulfate (HS) chains as a constant feature. They are expressed sequentially throughout mammalian neural development as progenitor cells progress through lineage differentiation.
7. Emerging biomimetic strategies are designed to mimic the interactions between HSPGs and key growth factors of neural lineage specification. These molecules may enable greater efficacy than natural HS GAGs in promoting neural differentiation, and are promising approaches for improving and advancing stem cell therapies for the treatment of neurological disorders.

## Introduction

Stem cells have the ability to self-renew through cell division and the capability to generate specialized cell types through differentiation under appropriate conditions (Fossett and Khan, 2012). Autologous (patient) or allogeneic (donor) transplantation of stem cells to regenerate tissue and repair damaged organs provide an exciting avenue for regenerative medicine. Neurological disorders encompass injuries or diseases of the nervous system, including neurodegenerative diseases and traumatic brain injuries. Effective regeneration of brain tissue requires damaged cells be replenished as well as their integration with existing cells to form new functional neural networks (van Velthoven et al., 2009). Injuries of the central nervous system (CNS) present therapeutic challenges as current treatments are unable to achieve complete functional recovery, even with symptomatic improvements (Neirinckx et al., 2013; Srijaya et al., 2014). To circumvent these limitations, a better understanding of the factors that control stem cell neural lineage specification is needed. In this review, we examine mechanisms within the neural niche influencing stem cell neural lineage potential, with a focus on the extracellular matrix heparan sulfate proteoglycans, and their role in stem cell neural fate determination.

## Stem cell neurogenesis

Neurogenesis is defined as the generation of functional neurons from neural precursors and is the first step in neuronal development, followed by axonal and dendritic guidance and synaptogenesis (Gage et al., 1998; Yamaguchi, 2001). Post-natal neurogenesis occurs throughout life in two selective neurogenic regions in the brain: the subventricular zone (SVZ) within the lateral ventricles, where resulting neurons migrate to the olfactory bulb (OB) through the rostral migratory system (RMS); and in the subgranular zone (SGZ) within the dentate gyrus of the hippocampus, where neurons ultimately migrate to the granular cell layer (GCL) to complete maturation (Zhao et al., 2008; Ming and Song, 2011; Gonçalves et al., 2016).

NSCs pass through a series of tightly regulated developmental stages governed by genetic, epigenetic and environmental signals before fully maturing to neurons (Urbán and Guillemot, 2014). During this process NSCs adopt specific marker expression profiles that have contributed to our ability to resolve the various stages of neuronal differentiation. These phenotypic markers are not sequential in their expression; rather they are transient overlapping markers expressed throughout lineage commitment (Kuhn et al., 2016). Although, these phenotypic markers provide a pattern with which to monitor neural development, their associated underlying molecular signaling events are currently not well understood.

In the event of insult to the brain, neurogenesis is triggered, resulting in production of neuroblasts that migrate from neurogenic niches to the site of injury. However, the inflammatory microenvironment present at the injury site is hostile to neuroblast survival, limiting the capacity of the brain for self-regeneration and repair (Galindo et al., 2011). In the case of neurodegeneration, the rate of cell loss exceeds that of generation of new neurons (Venugopal et al., 2017). With the balance of damage to regeneration unable to be met by the brain, obtaining neural cells from exogenous sources able to augment or direct repair is an important potential therapeutic tool (Neirinckx et al., 2013).

## Neural cell sources for therapeutic applications

Stem cell therapy is an emerging and promising approach for treatment of various neurological disorders through the transplantation of autologous or allogeneic stem cells via systemic infusion or local delivery (Law and Chaudhuri, 2013; Hao et al., 2014). Several stem cell-based therapeutic strategies may be applied to treating neurological disorders with stem cells able to enrich the NSC niche (enhance neurogenesis of endogenous NSCs) via the release of neuroprotective factors as well as becoming self-renewing neural progenitors (Sadan et al., 2009). With neurogenesis by NSCs following insult to the brain insufficient, obtaining neural cells from other sources is required. However, current models lack efficacy in the direction and control of stem cell neurogenesis to generate functioning neurons in adequate numbers for treatments (Sadan et al., 2009; Ma, 2010). Several studies examining transplanted stem cell migration to damaged areas have identified some success in terms of neuron function. This includes the use of murine NSC and MSCs in various traumatic brain injury and neurodegenerative disease models (Studer et al., 1998; Riess et al., 2002; Wang S. P. et al., 2012) as well as human NSCs (hNSCs) in murine stroke models (Jeong et al., 2003; Chu et al., 2004; Lee et al., 2007), supporting stem cell therapies in the treatment of neurological disorders (Kim and de Vellis, 2009; Assinck et al., 2017). In addition, neural progenitors and mature neural cells have been successfully generated from ESCs, iPSCs, NSCs, and MSCs (Kim and de Vellis, 2009).

### Neural stem cells (NSCs)

NSCs are isolated from the neurogenic regions of the adult brain (SVZ, SGZ), and from multiple regions of the embryonic brain. NSCs can be propagated *in vitro* as neurospheres or adherent cultures in serum-free media under high concentration of mitogens, such as fibroblast growth factor (FGF) and epidermal growth factor (EGF) (Gage, 2000). In culture, FGF-2 promotes NSC self-renewal and regulates neural progeny fate, with higher FGF-2 concentrations promoting the generation of glial cells and lower FGF-2 concentration producing cultures primarily of neurons (Yamaguchi, 2001). Differentiation protocols are now relatively routine through plating NSCs on extracellular matrix substances such as laminin to promote neural differentiation into neurons, astrocytes, and oligodendrocytes (Conti et al., 2005). Some consensus exists when characterizing differentiating NSCs, with the expression of the NSC marker nestin, neuronal lineage markers βIII-tubulin, MAP2, NeuN, and the astrocyte lineage marker GFAP commonly used to identify lineage potential of isolated and expanded cultures. Transplanted NSCs have been shown to survive in animal brain injury models and migrate to become region-specific cells, although only a small number of NSCs achieved this with a reported lack of neurogenesis observed (Gincberg et al., 2012; Rolfe and Sun, 2015). Challenges remain regarding the proliferation capacity of NSCs, likely due to the scarcity of hNSCs derived from surgical resections or post-mortem biopsies, as well as ethical issues surrounding the use of embryo-derived NSCs (Nam et al., 2015).

### Embryonic stem cells (ESCs)

ESCs are pluripotent cells originating from the inner cell mass of the blastocyst with high expansive potential and ability to give rise to cell lineages of all three germ layers (Zhang et al., 2001; Cai et al., 2008). ESCs are commonly induced to neural cell types *in vitro* through methods that recapitulate the embryonic neural development process (Abranches et al., 2009). This includes embryoid body (EB) formation in the presence of retinoic acid or conditioned media (Kurosawa, 2007); or through a monolayer culture system in the presence of FGF and notch ligands together with the bone morphogenetic protein (BMP) antagonist, noggin (Ying et al., 2003; Kunath et al., 2007). In a mouse temporal lobe epilepsy model, ESC-derived neural progenitor cells (NPCs) displayed enhanced survival and differentiation in the GCL when transplanted into the dentate gyrus (Venugopal et al., 2017). Interestingly, a study using an Alzheimer's disease mouse model has shown transplantation of undifferentiated ESCs led to extensive teratoma formation (Wang et al., 2006). This, combined with ethical and political issues surrounding the derivation of ESCs from embryonic tissue poses hurdles for their use in clinical practice (Venugopal et al., 2017).

### Induced pluripotent stem cells (iPSCs)

iPSCs are somatic cells reprogrammed to a pluripotent state via retroviral transduction of the same four transcription factors: OCT3/4, SOX2, Klf4, and c-Myc (Takahashi et al., 2007). Thus, iPSCs possess potential as an autologous source for treatment as well as to alleviate ethical concerns surrounding their use as they are easily derived from adult tissues (Compagnucci et al., 2014). iPSCs, commonly reprogrammed from fibroblasts, share similarities with ESCs in morphology, proliferation, gene expression, surface antigens and epigenetic profile, and like pluripotent cells they can differentiate into neurons and glial cells (Dolmetsch and Geschwind, 2011; Liu et al., 2013). However, tumorigenesis and genetic abnormalities of iPSCs have been reported, which must be addressed before they are safe for clinical use (Hunsberger et al., 2016; Nagoshi and Okano, 2017).

### Mesenchymal stem cells (MSCs)

MSCs are somatic stem cells commonly isolated from aspirates of the iliac crest bone marrow, although they can also be isolated from other tissues including dental pulp, umbilical cord blood, adipose tissue, trabecular bone and the placenta (DiGirolamo et al., 1999; Ma, 2010). Various groups including ours have shown MSCs to express neural genes, including nestin, glial fibrillary acidic protein (GFAP), and βIII–tubulin (TUBB3) in their undifferentiated state (Montzka et al., 2009; Foudah et al., 2013; Okolicsanyi et al., 2014, 2015). In addition, MSCs have been shown to migrate to the brain upon transplantation and differentiate into neuronal cells (Eglitis and Mezey, 1997). To date, there have been limited reports on the side effects of MSCs in clinical applications (Wagner et al., 2010), with several experimental studies reporting positive outcomes in the use of MSCs in the treatment of brain injury (reviewed elsewhere; van Velthoven et al., 2009). However, the number of transplanted stem cells to date has been small when compared with lesion injury volume and the portion of transplanted stem cells differentiating into neurons insufficient to adequately replace the damaged neural tissue (van Velthoven et al., 2009).

## The stem cell niche

The stem cell niche is described as the microenvironment that encompasses all the elements immediately surrounding stem cells when they are in their naïve state (Chen, 2010). The ECM, a major constituent of the stem cell niche, is a complex three-dimensional macromolecular structure directing spatiotemporal cues that influence stem cell behavior. Cellular components within the ECM contribute to its structure and remodeling reflecting its tissue specificity primarily through interactions with the cell surface, an active area where surface receptors interact with the ECM to modulate cellular processes (Kirn-Safran et al., 2009). Stem cells residing in the ECM influence cell signaling through secretion of ECM components, and these cells also receive and respond to critical biochemical and physical cues from the ECM (Chen, 2010). Traditionally the ECM was thought to provide structural support and strength to cells; however, increasing evidence demonstrates that the ECM has numerous active roles during development, morphogenesis and organogenesis, including facilitating cell adhesion, proliferation, migration, specification, differentiation, and survival (Tsang et al., 2010; Gattazzo et al., 2014). In addition, a variety of extrinsic factors in the neural ECM control stem cell self-renewal and differentiation, including transcription factors, growth factors, cytokines, epigenetic regulators, and non-coding RNAs (Mikami and Kitagawa, 2016). The dynamic and symbiotic relationship between the cell and ECM governs cell behavior. In order to overcome the challenges associated with the use of stem cells in neurological repair, identifying and harnessing the regulatory mechanisms underlying stem cell neural lineage differentiation is crucial.

Proteoglycans (PGs), a major constituent of the ECM, are comprised of a core protein to which several GAG chains covalently attach at specific sites (Esko et al., 2009). PGs are grouped by the GAG chains they carry and include the HSPGs, chondroitin sulfate proteoglycans (CSPGs), as well as the small leucine-rich-repeat proteins (SLRPs), all expressed in the CNS (Bulow and Hobert, 2006). PGs are centrally involved in several biological processes, including cellular growth, adhesion, migration, receptor binding, morphogen gradient formation, barrier formation, and interaction with other ECM components (Jones et al., 2002). These important proteins act as intermediaries, particularly those membrane-bound, and are often strategically located to receive environmental cues to directly influence intracellular signaling pathways. In stem cell differentiation, heparan sulfate (HS) GAGs appear to be more influential than chondroitin sulfate (CS) GAGs, potentially due to the presence of glucosamine residues in the HS sugar backbone. Pickford et al. demonstrated HS, and its highly sulfated analog heparin, to be more potent than CS in promoting neural phenotypes in mouse ESCs, likely due to chain length and structure (Pickford et al., 2011).

## Heparan sulfate (HS)

### HS biosynthesis and modification

The production of HS is not encoded by a single gene, but rather occurs during an elaborate posttranslational biosynthesis in the Golgi apparatus upon arrival of the core protein from the endoplasmic reticulum (Helledie et al., 2012). This process is initiated by the addition of an amino acid to a Glucuronic Acid-Galactose-Galactose-Xylose tetrasaccharide linker attached to the core protein via *O*-glycosylation of a serine residue. The tetrasaccharide linker is found on both HS and CS chains thus the attachment of the first N-acetylglucosamine (GlcNAc) residue is the commitment step of HS biosynthesis, a process catalyzed by exostosin-like 3 (Extl3) (Lindahl et al., 1998; Bernfield et al., 1999; Sarrazin et al., 2011). The enzyme complex composed of exostosin (EXT) family of enzymes, EXT1 and EXT2, performs HS chain polymerization through addition of alternating glucuronic acid (GlcA) and GlcNAc residues to the growing chain (Haupt et al., 2009; Sarrazin et al., 2011). The final HS chain length can vary over 10-fold dependent on cell type and the core protein, becoming 50–150 disaccharides in length once assembled (Bernfield et al., 1999).

After chain synthesis, successive modifications occur, beginning with replacement of N-acetyl groups of GlcNAc units with a sulfate group catalyzed by the *N*-deacetylase/*N*-sulfotransferase (NDST) family of enzymes. This is a partial process as NDSTs modify GlcNAc in clusters, leaving modified regions (N-sulfated, NS domains) interposed between unmodified regions (N-acetylated, NA domains) (Lindahl et al., 1998; Bernfield et al., 1999). NDSTs are responsible for the overall design of the HS chain as all subsequent modifications rely on the presence of the N-sulfoglucoamine residues (Grobe and Esko, 2002; Sugahara and Kitagawa, 2002). The next modification is C-5 epimerization of a GlcA immediately adjacent to an N-sulfoglucosamine unit to iduronic acid (IdoA), by C5 epimerase (C5-EP) (Sarrazin et al., 2011). A series of *O*-sulfation modifications then occur, beginning with iduronosyl 2-*O*-sulfotranferase (HS2ST), then glucosaminyl 6-*O* sulfotransferases (HS6ST) followed by glucosaminyl 3-*O* sulfotransferases (HS3ST), concluding the modification process in the Golgi apparatus (Bernfield et al., 1999; Sugahara and Kitagawa, 2002). Upon arrival at the cell surface or ECM, additional modifications may occur through 6-*O*-endo-sulfatases (Sulf1/2) and/or by the endoglycosidase heparanase (HPSE), further enhancing the heterogeneity and complexity of HSPGs (Sarrazin et al., 2011). It is through this structural diversity that these proteins selectively interact with a wide range of proteins, including FGFs, BMPs, and Wnts, and contribute to the regulation of stem cell behavior (Pulsipher et al., 2015).

### HS and neural development

HS is essential for mammalian brain development. This influence is generally accepted to occur through specific sulfation patterns, which serve as recognition elements for growth factors and other signaling molecules. As such, the biosynthesis and modification enzymes of HS are central to the final HS structure and its influence (Inatani et al., 2003; Gama et al., 2006). Expression of HS biosynthetic enzymes, including different isoforms, occur through spatiotemporal interactions. As a result, the HS profile of stem cells as they progress through various stages of differentiation are constantly undergoing modification (Kraushaar et al., 2013). Predictably, depletion of EXT1, NDST1/2, and selected HS2ST, HS6ST and Sulf isoforms have been demonstrated to result in brain abnormalities and malfunction in mouse models (Grobe and Esko, 2002; Inatani et al., 2003; McLaughlin et al., 2003; Pratt et al., 2006; Kalus et al., 2009). In general, when stem cells begin to differentiate the overall HS content within the microenvironment along with the processes regulating sulfation are active, indicated by increased expression of HS biosynthesis and modification enzymes (Forsberg et al., 2012; Tamm et al., 2012; Wang et al., 2017). In mouse ESCs (mESCs), 80% of the GAGs produced are HS when cells are in their undifferentiated state, with only ~30% N-sulfated (Smith et al., 2011; Kraushaar et al., 2013). Some increase in N-, 3-*O*-, and 6-*O*-, and 2-*O*-sulfation is observed when mESCs transition into neural lineages reflective of the conversion of neuroepithelial cells from a proliferative to neuronal differentiative state (Johnson et al., 2007).

Neurogenesis is regulated by a number of growth factors, with the association between HS and FGFs one of the most characterized interactions. FGF2 is known to promote NSC, MSC, and pluripotent stem cell proliferation (Dombrowski et al., 2009); and promotes phosphorylation of serine/threonine kinase (Akt) and MAP kinases, extracellular signal-regulated kinases (Erk1/2) and c-Jun N-terminal kinase (JNK), to control cell proliferation and survival (Yamada et al., 2017). For effective FGF signaling, FGF2 must bind to its high affinity FGF receptor (FGFR) with the lower affinity HS present (Yayon et al., 1991), forming the FGF-FGFR-HS complex in a 2:2:2 ratio in combination with FGFR dimerization (Gasimli et al., 2012). The presence of 2-*O*-sulfation in particular permits the binding of FGF2 to HS and subsequent Erk1/2 activation, with 6-*O*-sulfation required for inducing FGF2 activity via FGFR (Sugaya et al., 2008; Chan et al., 2015). A study by Stavridis et al. demonstrated for mESC neural specification to take place, FGF-induced Erk signaling is required for a discrete period of time, without this interaction, differentiation halts (Stavridis et al., 2007).

As cells switch from a proliferative state to lineage differentiation, increased 6-*O*-sulfation is accompanied by changes in FGF signaling and from a requirement of FGF2 to one for FGF1 (Brickman et al., 1998). The presence of 6-*O*-sulfation sites is regulated by isoforms of HS6ST and Sulf1/2, subsequently regulating the interaction between HS and a number of heparin-binding growth factors. Overexpression of the Sulfs results in reduced 6-*O*-sulfation and as demonstrated by Kalus et al. (2015); with Sulf knockout mice exhibiting brain development deficiencies, including reduced cell migration, survival and neurite outgrowth due to disruption of FGF2 and glial cell line-derived neurotrophic factor (GDNF) signaling (Kalus et al., 2009, 2015; Yamada et al., 2017). FGF4 is required during neural differentiation and is reduced in NDST1/2 double knockout mice, indicating FGF4 requires N-sulfation for signaling (Johnson et al., 2007). Wnt signaling, a crucial pathway in neural development, binds HS to exert its activity and is regulated by Sulf remodeling and 6-*O*-sulfation (Ai et al., 2003). During the later events of neuronal differentiation, HS has been demonstrated to play an essential role in synaptogenesis and neuronal excitability. Notably, removal of highly sulfated HS moieties by heparinase treatment leads to epileptiform bursts with long periods of inactivity due to impairment of long-term potentiation during synaptic transmissions (Korotchenko et al., 2014; Minge et al., 2017).

Contrary to the neuronal lineage, in the oligodendrocyte lineage, oligodendrocyte precursor cells (OPCs) carry HS chains with more 2-*O*- and 6-*O*-sulfation than mature oligodendrocytes, accompanied by higher transcript levels of HS2ST, NDST3, and C5-EP. FGF2 is the key growth factor in regulating OPC proliferation and maintenance, interacting with 2-*O*- and 6-*O*-sulfation sites (Properzi et al., 2008). HS sulfation alters as OPCs differentiate, with reduced N- and 6-*O*-sulfation biosynthesis accompanied by a reduced affinity for FGF2 (Stringer et al., 1999). Interestingly, we have demonstrated culture of embryo-derived hNSCs in astrocyte differentiation culture conditions results in up-regulation of NDST3 and HS6ST1, indicating an increased requirement for 6-*O*-sulfation in the astrocyte lineage, similar to observations in neuronal differentiation (Oikari et al., 2016b).

In addition, our group recently published evidence of HSPGs as novel markers of hNSC fate determination, one of only a few studies examining HSPGs in human cell models (Oikari et al., 2016b). In particular, when we examined HSPG biosynthetic machinery markers during neuronal differentiation of hNSCs, we observed up-regulation of EXT2, NDST2, NDST4, HS6ST2, and HS6ST3, suggesting an increasing reliance on 6-*O* sulfation, similar to EB formation from ESCs (Nairn et al., 2007). Interestingly, when we compared hNSCs with the more cortex-derived lineage-restricted normal human neural progenitor cells (nhNPCs), the nhNPCs were shown to expresses higher levels of C5-EP, NDST2, NDST4, HS6ST1-3, and HPSE but lower levels of EXT2 and HS2ST1. This suggests existing chains within the cellular microenvironment are modified from 2-*O* to 6-*O*-sulfation to accommodate the change in growth factor requirement (Oikari et al., 2016a).

## HSPGs and neurogenesis

HSPGs are comprised of a core protein with one or more HS side chains attached. HSPGs can be classed based on their cellular localization, with syndecans (SDCs) and glypicans (GPCs) membrane-bound; perlecan, agrin, and type XVIII collagen secreted into the ECM; and serglycin localized to secretory vesicles (Bernfield et al., 1999). SDCs, GPCs, and perlecan carry HS as a constant feature, with other HS carrying proteoglycans often decorated with other GAGs (Kraushaar et al., 2013). As most of our current understanding of HSPGs in neural development are derived from studies in rodents, distinct physiological and structure differences between human and rodent brains (reviewed in Oikari et al., 2014) suggest further studies of human neurogenesis is essential to advancing human neural stem cell therapies. Here we focus on the role of the SDCs, GPCs, and perlecan HSPGs in the context of neural lineage progression *in vivo* and *in vitro*.

### Membrane-bound HSPGs

**Figure 1:**
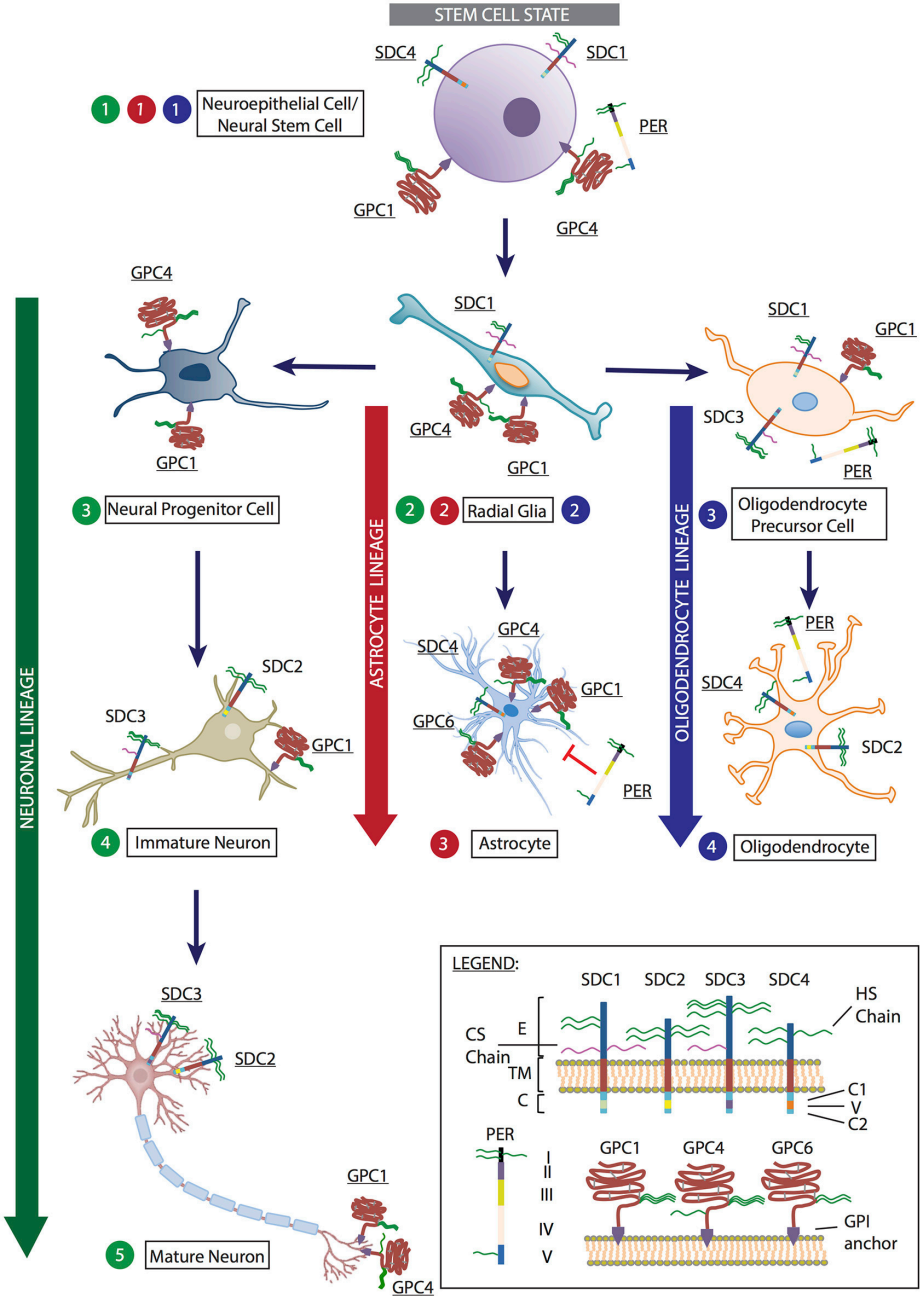
Representation of heparan sulfate proteoglycans (HSPGs) expressed by cells at various stages of neural stem cell differentiation into neuron, astrocyte and oligodendrocyte lineages. At the stem cell state (denoted in gray), neuroepithelial cells/neural stem cell (Step 1) expresses the HSPGs syndecan-1 (SDC1), syndecan-4 (SDC4), glypican-1 (GPC1), glypican-4 (GPC4), and perlecan (PER). These HSPGs mediate cell proliferation and maintenance of the pluripotent state via FGF and Wnt signaling. As cells begin to enter differentiation programs, they become radial glia (Step 2), with the potential to differentiate into the three main neural lineages: neurons, astrocytes and oligodendrocytes. At this stage, radial glia cells express SDC1, GPC1, and GPC4. Differentiation toward the neuronal lineage (denoted in green) results in the formation of neural progenitor cells (neuronal Step 3), which expresses GPC1 and GPC4. As cells progresses to immature neurons (neuronal Step 4), syndecan-2 (SDC2) is expressed on dendritic spines to promote dendritic maturation; syndecan-3 (SDC3) is localized to axons to assist axon guidance with GPC1 during synapse formation. This is also observed in mature neurons (neuronal Step 5) in conjunction with GPC4 mediation of excitatory synapse development through LRRTM4 binding. In astrocyte lineage differentiation (denoted in red), as radial glia cells differentiate toward astrocytes, SDC1 expression diminishes, SDC4 and glypican-6 (GPC6) expression becomes detectable while GPC1 and GPC4 expression is retained (astrocyte Step 3). The presence of PER is inhibitory to astrocyte proliferation due to the presence of domain V (astrocyte Step 3). In the oligodendrocyte lineage (denoted in blue), oligodendrocyte precursor cells (oligodendrocyte Step 3) derived from radial glia cells retain SDC1 and GPC1 expression, with these HSPGs assisting in cell proliferation and inhibiting differentiation. SDC3 and PER expression are up-regulated at this stage. As oligodendrocytes are formed (oligodendrocyte Step 4), SDC2 and SDC4 become the predominately expressed HSPGs, along with PER. (Inset) Legend: Syndecan-1-4 (SDC1-4). E, ectodomain; TM, transmembrane domain; C, cytoplasmic domain, including two conserved regions C1 and C2, and a variable region (V). Perlecan (PER) comprised of domains I-V. Glypican-1, −4, and −6 (GPC1, GPC4, and GPC6) are attached to the cell surface via a GPI-anchor. Astrocyte image obtained from: https://commons.wikimedia.org/wiki/File:Diagram_of_an_astrocyte_-_a_type_of_glial_cell_CRUK_029.svg. Mature neuron image modified from: https://online.science.psu.edu/bisc004_activewd001/node/1907.

SDCs are a family of type I transmembrane core proteins of the cell surface capable of carrying HS and CS chains, which are *O*-linked to a serine or threonine residue at the core protein (Xian et al., 2010). Four SDC genes exist in vertebrates (SDC1-4) and they all consist of an extracellular domain (ectodomain) at the N-terminal end, a hydrophobic transmembrane domain (TM), and a short cytoplasmic domain at the C-terminal (Lopes et al., 2006; Figure 1 inset). Through their cytoplasmic domains, the SDCs mediate transmembrane signaling of cytoskeletal and other signaling molecules. Interactions with PDZ-containing domains via their C2 region occurs through direct linkage to intracellular components, including the cytoskeleton and internal scaffolding (Rapraeger and Ott, 1998; Hsueh and Sheng, 1999). The ectodomain is specific for each SDC and contains several consensus sequences for GAG attachment (Carey, 1997).

During neural development, SDC1 has been shown to be highly expressed in the SVZ by neural precursor cells (NPCs) (including NSCs and radial glia cells) where it regulates proliferation and maintenance of NPCs (Figure 1, stem cell state, Steps 1 and 2). Wang Q. et al. identified disrupted canonical Wnt signaling in an SDC1 knockdown murine model due to reduced β-catenin, resulting in reduced cell proliferation and premature differentiation (Wang Q. et al., 2012). Also involved in early brain development, SDC4 expression can be detected at a reduced level in the ventricular zone (Ford-Perriss et al., 2003). SDC3 has been identified to play an essential role in promoting neuronal migration through binding GDNF via its HS chains to transduce GDNF signaling or by presentation of GDNF to its receptor tyrosine kinase, subsequently activating Src kinase (Bespalov et al., 2011; Poulain and Yost, 2015). SDC3 also acts as a receptor for heparin binding growth associated molecule (HB-GAM) in neurons to induce cytoskeletal changes via its C-terminal, thus playing a crucial role in the developing axon tract and promoting neurite outgrowth (Yamaguchi, 2001; Winkler et al., 2002; Figure 1, neuronal lineage, Steps 4 and 5). The conserved cytoplasmic domain of SDC2 contains four tyrosine residues, phosphorylated by Eph receptor tyrosine kinases to initiate dendritic spine morphogenesis (Ethell et al., 2001). Localization of SDC2 has been shown to be restricted to the synapses, where it contributes to dendritic spine formation, promoting polysialic acid-neural cell adhesion molecule-mediated synaptogenesis via its HS chains (Yamaguchi, 2001; Dityatev et al., 2004; Wang Q. et al., 2012), and accelerating synapse maturation via interaction with FGF22 (Hu et al., 2016; Figure 1, neuronal lineage, Steps 4 and 5).

Additionally, all SDCs are actively involved in the glial lineages. Oligodendrocyte progenitor cells (OPCs) expresses SDC1 and SDC3 where 2-*O* and 6-*O*-sulfated sites on the HS chains carried by SDC3 interact with FGF2 (Bansal et al., 1996; Winkler et al., 2002; Figure 1, oligodendrocyte lineage, Step 3). SDC3 also plays a role in OPC migration where it acts as a co-receptor for FGF2 and as a receptor for HB-GAM (Winkler et al., 2002). As OPCs differentiate to mature oligodendrocytes, SDC2 and SDC4 are the predominantly expressed SDCs (Bansal et al., 1996; Properzi et al., 2008; Figure 1, oligodendrocyte lineage, Step 4). SDC4 is the only SDC reported to be expressed in rat astrocytes (Avalos et al., 2009; Figure 1, astrocyte lineage, Step 3).

GPCs are a family of HSPGs attached to the exocytoplasmic surface of the plasma membrane via a glycosylphosphatidyl-inositol (GPI) anchor (Feng et al., 2012). Six GPC family members can be found in mammalian cells (GPC1-6) comprised of the characteristic pattern of 14 highly conserved cysteine residues, located near the N-terminus or central domain of the core protein (Fransson, 2003). These residues are thought to form intramolecular disulfide bonds, giving GPCs a conserved globular tertiary structure (Song and Filmus, 2002; Fico et al., 2011; Figure 1 inset).

**Figure 2:**
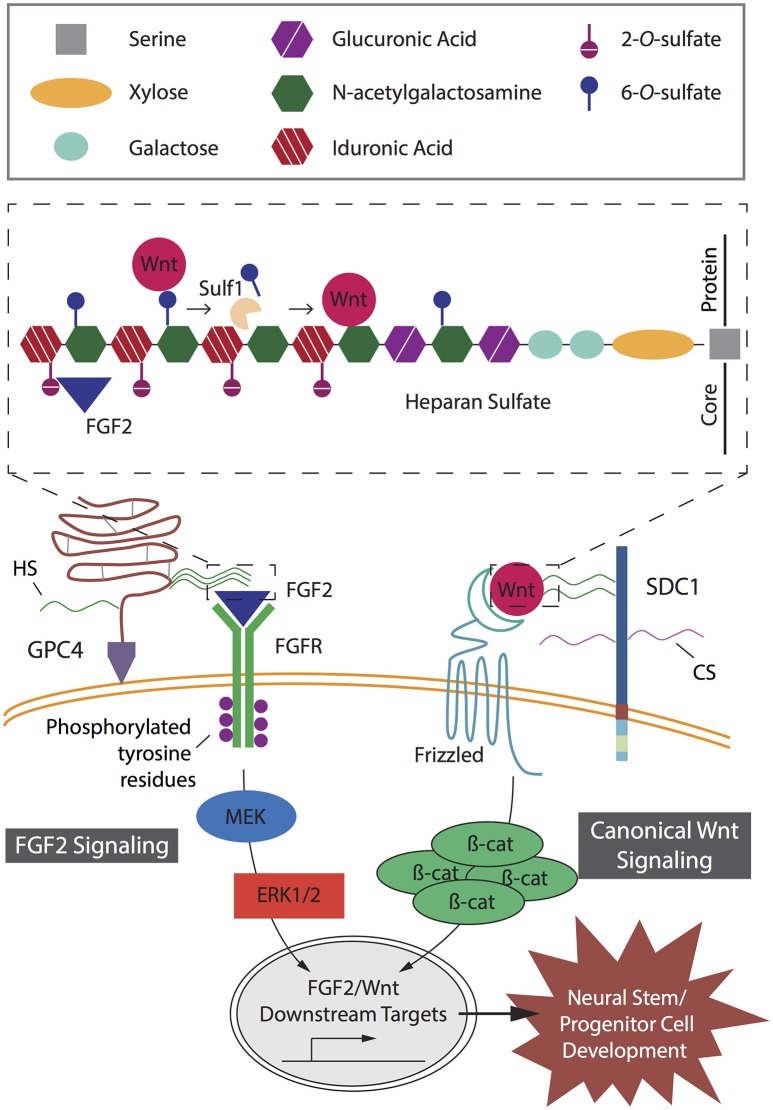
Key heparan sulfate proteoglycan (HSPG)-mediated signaling pathways in neural stem and progenitor cell (NSPC) development. Fibroblast growth factor (FGF2) and canonical Wnt signaling pathways act independently to mediate proliferation and lineage differentiation of cells with specific interactions determined by specific heparan sulfate chain sulfation profiles. In FGF2 signaling, GPI-anchored HSPG glypican-4 (GPC4) HS chains modulate the binding of FGF2 to its receptor, FGFR. Binding of FGF2 to HS requires 2-*O*-sulfates (inset). Subsequent phosphorylation of tyrosine residues mediates interactions with cytosolic adaptor proteins; resulting in activation of the MEK/ERK cascade and downstream targets, key transcriptional factors promoting NSPC development. In canonical Wnt signaling, the Wnt ligand binds to its receptor, Frizzled, and is mediated by SDC1 via 6-*O*-sulfation sites on the HS chains (inset). The presence of HS chain 6-*O*-sulfation sites enables high affinity binding of Wnt ligands and prevents interaction between Wnts and their Frizzled receptors. In the presence of Sulf1, selective 6-*O*-sulfates are removed from the HS chain, resulting in low affinity binding of Wnt ligands to the 6-*O*-desulfated site allowing presentation to the Frizzled receptors. Wnt binding to its receptor leads to accumulation of β-catenin in the cytoplasm, which translocates into the nucleus to activate downstream targets and promote NSPC development.

GPC4 is most predominantly expressed during mouse neural development in the ventricular zone of the telencephalon, and acts as a modulator of FGF2 to maintain NSC and NPC proliferation and self-renewal (Hagihara et al., 2000; Yamaguchi, 2001; Ford-Perriss et al., 2003). GPC4 appears to be a marker of stem and progenitor cells (Figure 1, stem cell state, Steps 1 and 2; neuronal lineage, Step 3), with its expression lost as cells commit to neural lineage differentiation and maturation (Figure 1, neuronal lineage, Steps 4 and 5), as observed in rodents *in vivo*, NSCs *in vitro* (Hagihara et al., 2000; Ford-Perriss et al., 2003; Gasimli et al., 2012), and mouse ESCs (Fico et al., 2012). Interestingly, various group have shown leucine-rich repeat transmembrane proteins (LRRTM) to be important regulators of excitatory synaptogenesis *in vivo* and in cultured rodent dentate granule neurons. HSPGs, particularly GPC4, were found to be novel presynaptic receptors of LRRTM, binding preferentially to postsynaptic LRRTM4 and not LRRTM2 via HS chains (de Wit et al., 2013; Siddiqui et al., 2013; Figure 1, neuronal lineage, Step 5). Additionally, protein tyrosine phosphatase sigma (PTPσ) is a presynaptic receptor for the GPC4/LRRTM4 complex, with the HS-binding ability of PTPσ essential for excitatory synapse transmission (Ko et al., 2015). Jen et al. demonstrated a significant reduction in brain size in GPC1-null mice, attributed to FGF17 signaling and reduced cell proliferation (Jen et al., 2009) with GPC4 and GPC1 appearing to share overlapping functions in regulating cell proliferation and brain size. Interestingly, SDC1, which also regulates neural stem and progenitor cell (NSPC) proliferation, functions through the Wnt pathway with SDC1 knock down models showing no interference with FGF signaling (Poulain and Yost, 2015; illustrated in Figure 2); with GPC1 expression observed in mature neurons localized to the axons and nerve terminals (Litwack et al., 1998; Yamaguchi, 2001; Jen et al., 2009; Figure 1, neuronal lineage). These examples demonstrate the diversity and complexity of HSPGs in regulating multiple signaling pathways to coordinate neural development. Our findings in hNSCs are comparable to the mouse model where GPC1 knockdown reduced the expression of the NSC marker nestin, and neuronal markers TUBB3 and NEFM, further supporting a key role for GPC1 in NSC proliferation and neuronal differentiation (Oikari et al., 2016b). GPC5 and GPC2 have also been identified to be expressed in post-mitotic neurons, and in axon tracts respectively (Litwack et al., 1998; Yamaguchi, 2001).

During gliogenesis, both OPCs and oligodendrocytes express GPC1 (Figure 1, oligodendrocyte lineage), likely due to its interaction with FGF2, a crucial mitogen for OPCs to stimulate proliferation and inhibit differentiation. As OPCs differentiate, the HS chains of GPC1 are modified to alter their FGF2 binding capability (Winkler et al., 2002). Astrocytes in the developing rat CNS express GPC1, GPC4, and GPC6 (Winkler et al., 2002; Allen et al., 2012; Figure 1, astrocyte lineage). Similarly, when we examined hNSCs in astrocyte lineage culture conditions, we also observe an up-regulation of GPC4 localized to specific regions when examined by immunocytochemistry. Interestingly, siRNA knockdown of GPC4 in hNSC cultures results in down-regulation of the astrocyte marker S100B, supporting a role for GPC4 in mediating the astrocyte lineage (Oikari et al., 2016b).

Our work in hNSCs has also demonstrated that as the cells differentiate toward the neuronal lineage, SDC4, GPC1, GPC2, GPC3, and GPC6 are all up-regulated. Interestingly, expression of these genes was observed to be lower than the level observed in the more neuronal lineage-restricted nhNPCs, where higher gene expression levels of SDC2, 3, 4 as well as GPC1, 2, 3, and 6 were observed, similar to murine studies (Oikari et al., 2016a,b). As cell surface-bound HSPGs, SDCs, and GPCs play an important role in promoting neurogenesis and may provide additional markers for defining markers of neural cell types during lineage specification.

### Matrix-localized HSPGs

During mammalian neural development, the HSPG perlecan is expressed in the basal lamina of the neuroepithelium, a crucial component of the neural niche (Ford-Perriss et al., 2003; Figure 1, stem cell state, Step 1). Perlecan is a multi-domain HSPG of the ECM with diverse roles during development and organogenesis (Knox and Whitelock, 2006). A large protein with five domains, perlecan carries 3-4 HS chains in domain I and one in domain V (Figure 1 inset), which interact with heparin-binding growth factors such as FGF-2, EGF, and VEGF (Farach-Carson and Carson, 2007). Perlecan is another multifunctional protein in the developing mouse brain, where it promotes NSPC proliferation in the SVZ by acting as a co-receptor for FGF2 (Nurcombe et al., 1993; Yamaguchi, 2001; Girós et al., 2007). FGF2 is essential in the mouse NSC niche, and requires perlecan to promote proliferation through activation of the Akt and Erk1/2 pathway, elegantly demonstrated by Kerever et al. (2014) in a perlecan null murine model. In the same study, FGF2 failed to promote neurosphere formation of GFAP^+^CD133^+^ NSCs owing to the inability to induce cell cycle progression via cyclin D2 (Kerever et al., 2014). In another study by Girós et al. (2007), perlecan-null mice displayed impaired forebrain development, through disrupted sonic hedgehog (Shh) signaling, with perlecan essential to mediate the Shh concentration gradient (Girós et al., 2007; Palma et al., 2011).

*In vitro* studies by Nakamura et al. (2015) demonstrated addition of exogenous perlecan to neural cultures promoted NSPC proliferation as well as neurite extension. Addition of heparin was found to promote NSPC proliferation, although this was not significant, suggesting perlecan as well as its associated GAGs are required. Perlecan has also been demonstrated to play a role in gliogenesis, where OPCs show increasing expression of perlecan during terminal differentiation of mature oligodendrocytes (Winkler et al., 2002; Figure 1, oligodendrocyte lineage). In contrast, perlecan expression in astrocytes has been shown to inhibit proliferation due to domain V (Figure 1, astrocyte lineage, Step 3), with heparin also shown to a lesser extent to suppress astrocyte proliferation (Nakamura et al., 2015). With its contribution to differentiation of the astrocyte and oligodendrocyte lineages, perlecan is likely a marker for glial lineage specification.

## Approaches for exploiting HSPGs as therapeutic targets

The emerging evidence of a central role for HSPGs in regulating neural stem cell lineage fate may provide opportunities to better understand and control lineage specification and to fully exploit the use of stem cells as effective therapeutics. With HSPGs involved at all stages of stem cell maintenance and neurogenesis, including proliferation, self-renewal, differentiation, migration, and maturation, multiple opportunities exist to develop improved therapeutics.

**Figure 3:**
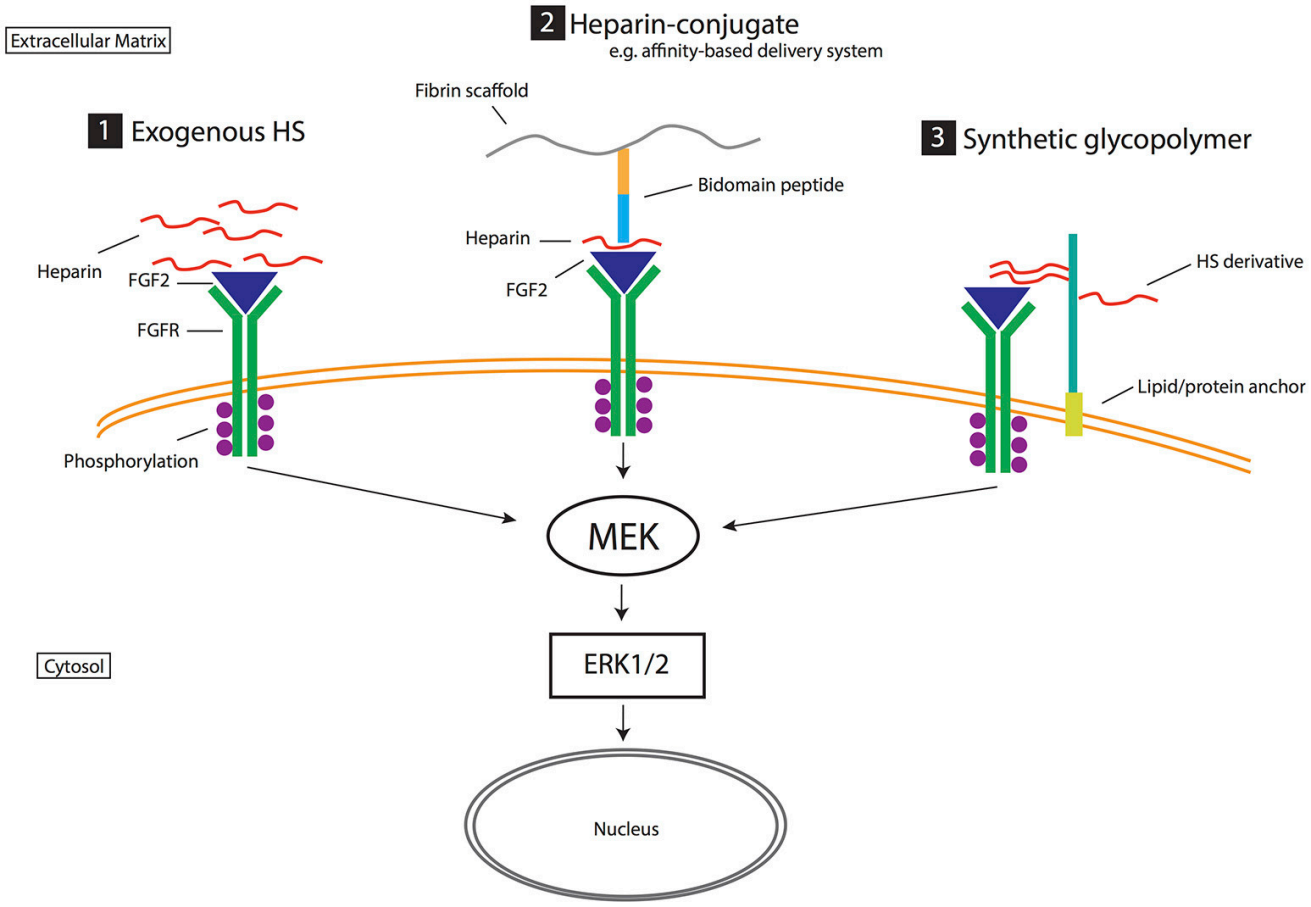
Biomimetic strategies for exploiting heparan sulfate proteoglycans and their role in stem cell neurogenesis. Strategy (1) Exogenous HS. Heparin is a highly sulfated analog of HS that promotes growth factor activity such as stabilizing the binding of FGF2 to FGF receptor (FGFR). Strategy (2) Heparin-conjugate. Heparin is linked to various biomaterials to enhance its ability to mediate growth factor and receptor interactions (FGF2 activity in this example). For example, in the affinity-based delivery system (Willerth et al., 2008) heparin is bound to both a bidomain peptide linked to fibrin scaffolding and heparin-binding growth factor (i.e., FGF2). This technique harnesses the ability of heparin to bind growth factors, prevent degradation and potentiate receptor binding via a controlled release mechanism. Strategy (3) Synthetic glycopolymer. HS derivatives/synthetic HS are introduced to the cell surface via lipid or protein anchors, mimicking the role of HS in mediating growth factor signaling (e.g., FGF2 signaling). These strategies target heparan sulfate and growth factor interactions. In FGF2 signaling, extracellular signal-regulated kinases (Erk1/2) controls cellular proliferation and differentiation. Affinity-based delivery system sketch is modified from Willerth et al. (2008).

Stem cell fate can be directed through exogenous and synthetic HSPGs and GAGs. Exogenous heparin, the highly sulfated analog of HS, is a widely utilized means of enhancing HS-mediated stem cell proliferation and self-renewal (Figure 3; Fossett and Khan, 2012). The study performed by Pickford et al. (2011), identified the heparin effect is dependent on size (length) and concentration (Pickford et al., 2011), and varies between cell culture systems with sensitivity to heparin observed to differ between hMSC and hESC cultures (Mimura et al., 2011). In order to enhance the effect of heparin, a popular strategy is to conjugate heparin with assorted biomaterials (Figure 3; van Velthoven et al., 2009 and reviewed in Sakiyama-Elbert, 2014). The Sakiyama-Elbert group (2014) devised an affinity-based delivery system through the combination of a biomaterial, heparin and growth factors. They suggest this conjugation enhances the capability of heparin to bind a wide range of growth factors, preventing degradation and potentiating receptor binding via a controlled release mechanism (Willerth et al., 2008).

Harnessing HSPG and growth factor interactions has also resulted in various forms of HS mimetics and synthetic glycopolymers that mimic natural occurring HSPGs and their function during stem cell neural differentiation. These strategies have produced synthetic glycopolymers with even higher efficacy than heparin (Figure 3; Neirinckx et al., 2013). In other studies, Liu et al. (2017) devised a phospholipid-anchored GAG-mimicking polymer, termed “lipo-pSGF,” found to promote neural differentiation, likely via the FGF2-Erk1/2 pathway, in mESC cultures and have compared its efficacy to non-anchored pSGF and heparin treated cultures. The lipid-anchor maintained the lipo-pSGF on the cell surface without being exocytosed (Liu et al., 2017). In another variation, Huang et al. (2014) generated synthetic neoproteoglycans (neoPGs) that also promoted neural differentiation of mESCs via the FGF2/Erk pathway (Huang et al., 2014). Similarly, Pulsipher et al. covalently attached GAG derivatives to the cell membrane of mESCs via a HaloTag protein (HTP) anchor, which provided a longer (more than a week), stable presentation of defined HS GAGs when compared to lipid-anchoring (Pulsipher et al., 2015). Through both the HTP anchor and defined sulfation pattern on the HS derivative, the FGF/Erk pathway was activated to promote enhanced exit of mESCs from self-renewal to differentiation of neuronal cell types (Figure 3). Despite these promising data, there is no current compound that fully mimics natural HSPGs (Pulsipher et al., 2015). However, synthetic GAGs have some advantages over natural GAGs, and provide the opportunity to synthesize compounds of structural homogeneity, purity, and controlled sulfation to circumvent any limitations (Wang et al., 2015, 2017).

## Conclusion and perspective

Despite no current long-term/permanent therapies, stem cells have potential in cell/tissue replacement therapies for neurological disorders. Successful therapies require regimens for differentiating stem cells into desired lineages with high efficiency and yield. Current approaches include combinations of growth factors and chemicals, which often correlate with relatively poor yield of desirable cell types, and poor therapeutic efficacy. To overcome these challenges, a comprehensive understanding of the extrinsic and intrinsic signals regulating neural stem cell fate must be defined.

Identification of specific HSPGs and HS sulfation patterns as they are expressed throughout neural lineage progression will allow for distinction between neuronal, astrocyte and oligodendrocyte lineage specification and enhance derivation of these cell types from the stem cell state. HS sulfation motifs are specific for each cell type and it is essential to characterize HS structural changes in specific stem cell models. Further research, particularly in the human biological system, is required to identify point/s of intervention during neurogenesis. HS biomimetics to mimick HSPG core proteins and/or specific HS moieties are promising strategies for enriching current stem cell therapies, with more rigorous analysis required for their routine, reproducible and safe integration into therapeutic applications. Forthcoming work examining the intrinsic mechanisms governing the influence of HSPGs in mediating neural stem cell fate will likely enable better cell isolation techniques, as well as the development of tailored production of specific neural cell types for various applications and treatment requirements, and promote advances in biomimetic engineering.

## Author contributions

CY developed the concept and drafted the manuscript. LH conceived of the manuscript, supervised the structure and design of the manuscript, revised the manuscript, and approved the final version. LG revised the manuscript and approved the final version.

### Conflict of interest statement

The authors declare that the research was conducted in the absence of any commercial or financial relationships that could be construed as a potential conflict of interest.
